# Social rank-associated stress vulnerability predisposes individuals to cocaine attraction

**DOI:** 10.1038/s41598-018-19816-x

**Published:** 2018-01-29

**Authors:** Chen Yanovich, Michael L. Kirby, Izhak Michaelevski, Gal Yadid, Albert Pinhasov

**Affiliations:** 1 Department of Molecular Biology, Ariel University, Ariel, Israel; 2 Leslie and Susan Gonda (Goldschmied) Multidisciplinary Brain Research Center and the Mina and Everard Goodman Faculty of Life Sciences, Bar-Ilan University, Ramat-Gan, Israel

## Abstract

Studies of personality have suggested that dissimilarities in ability to cope with stressful situations results in differing tendency to develop addictive behaviors. The present study used selectively bred stress-resilient, socially-dominant (Dom) and stress-vulnerable, socially-submissive (Sub) mice to investigate the interaction between environmental stress and inbred predisposition to develop addictive behavior to cocaine. In a Conditioned Place Preference (CPP) paradigm using cocaine, Sub mice displayed an aversion to drug, whereas Dom mice displayed drug attraction. Following a 4-week regimen of Chronic Mild Stress (CMS), Sub mice in CPP displayed a marked increase (>400%) in cocaine attraction, whereas Dom mice did not differ in attraction from their non-stressed state. Examination of hippocampal gene expression revealed in Sub mice, exposure to external stimuli, stress or cocaine, increased CRH expression (>100%), which was evoked in Dom mice only by cocaine exposure. Further, stress-induced decreases in DRD1 (>60%) and DRD2 (>50%) expression in Sub mice differed markedly from a complete lack of change in Dom mice. From our findings, we propose that social stratification dictates vulnerability to stress-induced attraction that may lead to addiction via differential regulation of hippocampal response to dopaminergic input, which in turn may influence differing tendency to develop addictive behaviors.

## Introduction

Ready availability of a variety of narcotic substances coupled with abundant 21-century stressors has markedly increased drug use and produced accompanying social problems of a grave nature^1,2^. There is literature evidence of the significant association between personality, stress and the motivation to abuse addictive substances^3–5^. The ability of individuals to cope effectively with stress significantly predicts whether a person will develop drug addiction^4^.

Results of personality studies in humans and animal models have suggested that differences exist with respect to stress responses based on coping style^6^. Socially dominant individuals display a stress resilience, typified by proactive behaviors^7,8^. One such proactive behavior is impulsiveness, which has been linked as a vulnerability factor to substance abuse in both clinical and preclinical studies^9^. In contrast, subordinate individuals are more prone to develop depression^10,11^ and under conditions of stress from trauma or social defeat have a tendency to engage in social avoidance behaviors and migrate to self-indulgent coping styles such as substance abuse^12^. The underlying drivers of these baseline coping strategies and responses have been assumed to reside within functioning of the limbic system, prominently mediated by baseline mesolimbic dopaminergic tone, with impulsive individuals displaying low baseline dopaminergic output and non-impulsive individuals displaying high baseline dopaminergic output^13^. Not to be excluded, the hippocampus and associated memory circuits also play an important role in reinforcement of memories, responses to stressful situations and successful coping strategy decision outcomes to guide future behavioral responses^14^.

The hippocampus is important for drug-associated memory cues established from prior drug use. Functional magnetic resonance imaging (fMRI) and positron emission tomography (PET) studies have revealed that hippocampal activity in drug users is altered. Environmental cues such as cocaine use and drug preparation boost hippocampal glucose metabolism^15^ and activation^16^. These changes are functionally-relevant since videos of drug use cues also activate downstream hippocampal targets such as amygdala and cingulate gyrus^17^. Hippocampal activation in response to drug cues appears to be dependent on increase dopamine secretion in CA1 region^18^ and is dependent on DRD1/DRD5 receptors driving establishment of novel stimuli-based memories through a pathway originating from the locus coeruleus^19^. Therefore, any system that influences expression of hippocampal dopamine receptors or dopamine secretion in response to drug use is likely to act as a reinforcer of drug use memories.

In the present study we explored reaction to cocaine in naïve state and under stress using unique selectively-bred mice with strong and stable features of dominance (Dom) and submissiveness (Sub) that represent robust resilience or vulnerability to stress respectively^20–22^. These selectively bred strains were well characterized previously by markedly differing stress-related behavioral and hormonal reactions and differential response to antidepressants and mood stabilizing agents^20^. In addition, we report differential regulation of hippocampal genes relating to memory encoding under conditions of stress or drug exposure between stress vulnerable and resilient mice and propose an underlying mechanism of stress vulnerability resides in differences in the encoding of memories. Anxiogenic effects are known to be evoked by increased hippocampal CRH receptor 1 (CRHR1) stimulation in the CA1 subfield. Further, hippocampal CRH has a strong influence on context-dependent fear conditioning and aversive behaviors, and its increased expression is known to selectively disrupt dopaminergic regulation of the hippocampus by a phospho-CREB-dependent mechanism^23^. CRHR activation is known to strongly influence stress-induced return to addictive behavior, presumably through reactivation of drug-associated memories and limbic reinforcement^24^.

Here, we postulate that behavioral differences in stress sensitivity with respect to drug attraction are mediated through differences in CRH-dependent hippocampal pyramidal cell activation and subsequent effects on hippocampal dopamine receptor expression. Stress-resilient individuals do not exhibit stress-induced hippocampal changes, whereas stress-prone individuals do present with hippocampal changes which thus reflects a contrast in processing of salient stimuli relating to drug exposure and affects both drug attraction and drug use reinstatement.

## Results

### Dom and Sub mouse models: selective breeding and stable dominant and submissive behavior

**Figure 1:**
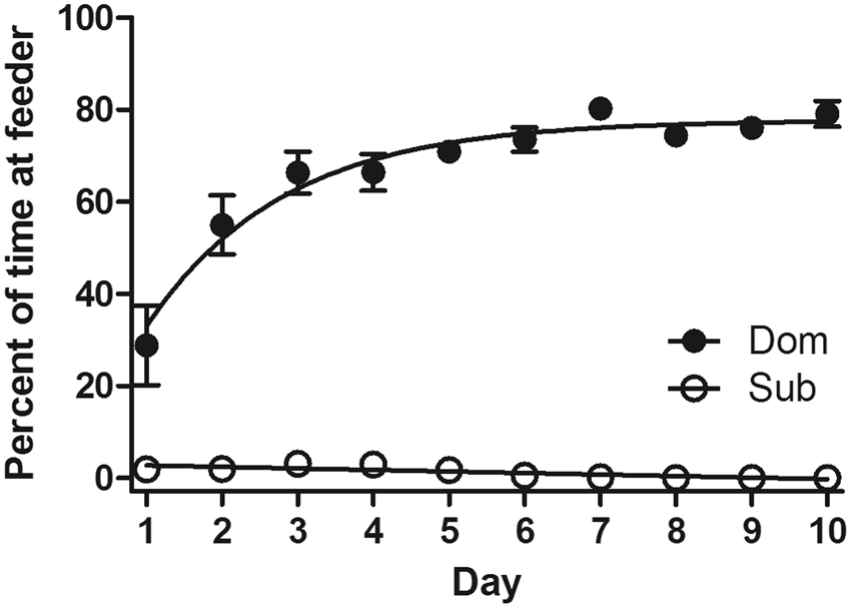
DSR results at generation 32 of Dom and Sub strains are stable and predictable. Data expressed as mean (±SEM) percent time spent at feeding well. Bonferroni means separation test indicated differences between mouse strains on all days (p < 0.001).

Selective breeding for dominant and submissive behavioral traits has produced two mouse strains with over 98% positive DSR response^20^. As seen in Fig. 1, 32^nd^ generation Dom mouse feeding time markedly differs from their paired Sub counterparts on a consistent basis (Two-way Repeated Measures ANOVA: Day: F[9,162] = 13.14, p < 0.0001; Strain effect: F[1,18] = 1207, p < 0.0001; Interaction: F[9.162] = 15.49, p < 0.0001).

### DOM and SUB animals show differential reaction to cocaine in the CPP test

**Figure 2:**
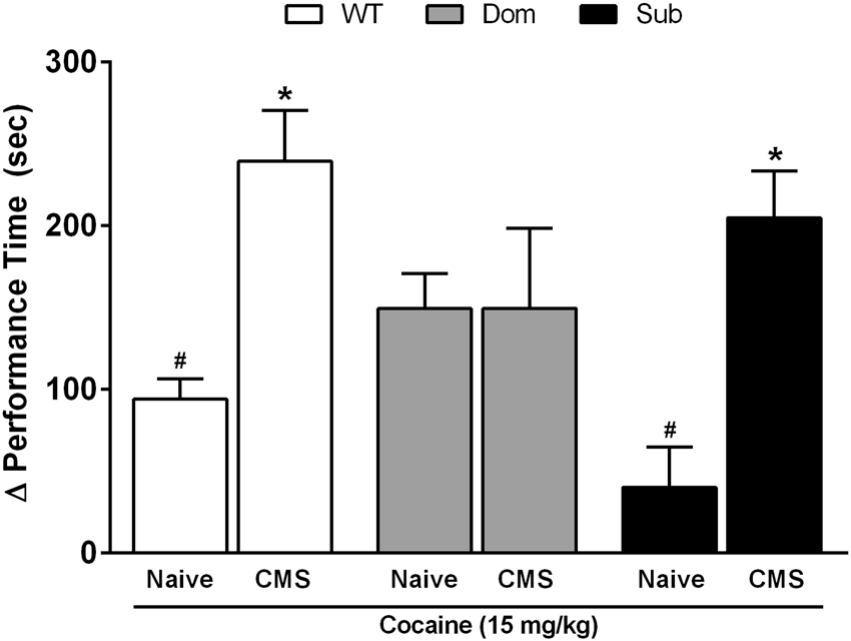
CPP performance time at day 6 in cocaine-treated mice (15 mg/kg): stress-naïve or 4-week CMS-exposed. Two-way ANOVA (F[1,35] = 21.70, p < 0.0001) with Bonferroni means separation tests: ^#^strain differences among naïve treatments; ^*^within stain differences between naïve and CMS treatments. Data are corrected to the difference (Δ performance time [s]) from day 0 drug-side baseline preference of individuals.

Stress-resilient (Dom), stress-sensitive (Sub), and reference strain (wild-type, Sabra) mice were examined in a CPP paradigm under conditions of CMS and/or cocaine exposure. In the stress-naïve state, Dom mice displayed higher attraction to drug relative to both WT (1.6-fold increase over WT) and Sub (3.7-fold increase over Sub) mice (Fig. 2; two-way ANOVA: Interaction F[2,35] = 5.564, p = 0.0075; CMS effect F[1,35] = 21.70, p < 0.0001; Strain effect F[2,35] = 1.312, p = 0.2823). Whereas WT and Sub mice displayed moderate attraction to cocaine during stress-naïve state, attraction to drug markedly increased following a 4-week regimen of CMS (WT: 2.5-fold; Sub: 5.1-fold). In contrast, Dom mice were behaviorally refractory to the effects of CMS and did not display increased preference for drug. Due to the marked contrast in cocaine attraction and behavior observed among strains, these treatment protocols were retained for further study of associated changes in gene expression.

### Hippocampal *CRH* expression is upregulated by cocaine treatment, but *CRH* receptor expression is downregulated by chronic mild stress

**Figure 3:**
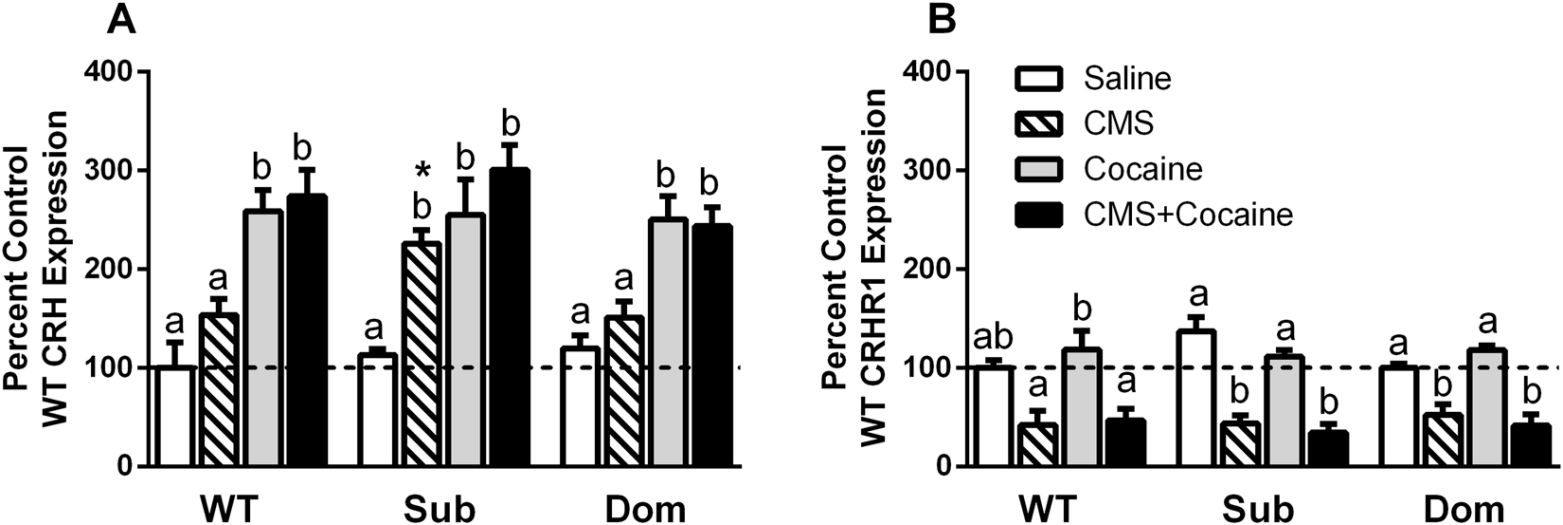
Effect of CMS and cocaine on Hippocampal *CRH* expression. Mice were exposed to CMS or no stress for 4 weeks, then treated with cocaine (15 mg/kg) or saline for 1 week in the CPP protocol. Letters above bars: one-way ANOVA with Bonferroni means separation test comparing treatments within mouse strain [*abc*] or treatments between strains [*]. (**A)** Corticotropin releasing hormone (*CRH*: WT: [F(3,16) = 13.34, p = 0.0001]; Sub: [F(3,16) = 11.83, p = 0.0002]; Dom: [F(3,16) = 12.63, p = 0.0002]; CMS: [F(2,12) = 7.219, p = 0.0087]). (**B)** *CRH* receptor 1 (*CRHR1*: WT: [F(3,16) = 7.596, p = 0.0022]; Sub: [F(3,16) = 26.11, p < 0.0001]; Dom: [F(3,16) = 19.39, p < 0.0001]).

We examined the effects of stress and cocaine on hippocampal *CRH* expression since CRHR1 activation on pyramidal neurons in hippocampus has been shown to reinforce stress- and drug-associated memories. *CRH* mRNA levels in Dom and Sub mice hippocampi increased similarly in response to cocaine exposure (Fig. 3A; one-way ANOVA within strain: WT: F[3,16] = 13.34, p < 0.0001; Sub: F[3,16] = 11.83, p = 0.0002; Dom: F[3,16] = 12.63, p = 0.0002). *CRH* receptor mRNA expression, however, was downregulated equally across strains by CMS (Fig. 3B; one-way ANOVA within strain: WT: F[3,16] = 7.596, p = 0.0022; Sub: F[3,16] = 26.11, p < 0.0001; Dom: F[3,16] = 19.39, p < 0.0001). Comparisons of treatments across strains indicated that stress altered CRH expression in a strain-dependent manner with Sub mice showing increased CRH expression (Fig. 3A; one-way ANOVA: F[2,12] = 7.219, p = 0.0087). The increase in *CRH* gene expression (ca. 2-fold) in response to CMS observed in the Sub strain, increases which approximated that observed with cocaine alone Glucocorticoid receptor and brain-derived neurotrophic factor mRNA expression did not change in response either to drug or stress (data not shown).

### DOM mice refractory to stress-related behaviors after CMS also lack stress-induced *DRD1* downregulation effect in hippocampus

**Figure 4:**
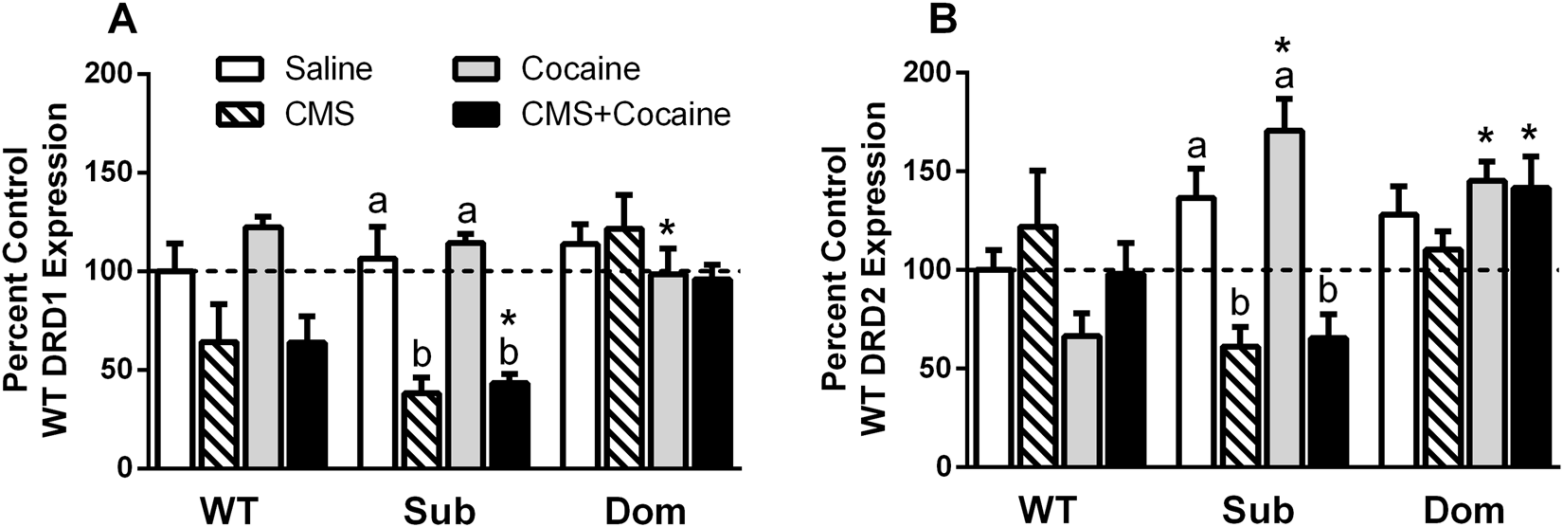
Effect of CMS and cocaine on locus coeruleus-hippocampal tract activity. Mice were exposed to CMS or no stress for 4 weeks, then treated with cocaine (15 mg/kg) or saline for 1 week in the CPP protocol. Letters above bars: one-way ANOVA with Bonferroni means separation test comparing: treatments within mouse strains [*abc*] or treatments across mouse strain [*]. (**A)** Dopamine receptor 1 (*DRD1*: WT: [F(3,16) = 4.303, p = 0.0209]; Sub: [F(3,16) = 17.43, p < 0.0001]; CMS: [F(2,12) = 7.49, p = 0.0077; CMS + Drug: [F(2,12) = 8.431, p = 0.0052]). (**B**) Dopamine receptor 2 (*DRD2*: Sub: [F(3,16) = 15.89, p < 0.0001]; Drug: [F(2,12) = 18.02, p = 0.0002; CMS + Drug: [F(2,12) = 6.695, p = 0.0111]).

Hippocampal dopamine receptor activation, particularly DRD1/DRD5, is known to play a critical role in reinforcement of stress-based behaviors and opioid addiction, whereas DRD2 receptors function in a feedback modulatory role for dopamine release. We therefore examined changed in DRD1 expression following chronic stress or cocaine exposure in Dom, Sub and WT mice to determine if either stimulus or both in combination produced any differential, strain-dependent responses that may relate to relative degrees of stress vulnerability. *DRD1* receptor (excitatory) mRNA was consistently downregulated (WT 36.0%, Sub 68.2%) in response to CMS exposure in both Sub and WT strains, however no *DRD1* stress effect was observed in Dom mice (Fig. 4A; one-way ANOVA within strain: WT: F[3,16] = 4.303, p = 0.0209; Sub: F[3,16] = 17.43, p < 0.0001). When *DRD2* (inhibitory) mRNA expression was examined, only Sub mice responded with CMS-mediated downregulation (ca. 45–50%, Fig. 4B; Sub: F[3,16] = 15.89, p < 0.0001), whereas Dom and WT strains were not responsive (Fig. 4B). Comparison of treatment effects across strains indicated that stress produced marked decreases in DRD1 expression in Sub mice compared with Dom mice (Fig. 4A; one-way ANOVA: Stress: F[2,12] = 7.490, p = 0.0077; Stress + Cocaine: F[2,12] = 8.431, p0.0052). Cocaine produced upregulation of DRD2 expression in Sub and Dom mice compared with WT (Fig. 4B; one-way ANOVA: F[2,12] = 18.02, p = 0.0002), whereas combination of stress and cocaine produced a downregulation of DRD2 expression in Sub mice compared with Dom mice (Fig. 4B; F[2,12] = 6.695, p = 0.0111). *CRH* was inversely correlated with *DRD1* expression across all groups examined (R_S_ = −0.231, df = 58, p = 0.0379).

## Discussion

In the present study, mice with strong dominant and submissive behavior exhibiting resilience and sensitivity to stress, respectively, were found to display marked differences in cocaine attraction and CMS-mediated regulation of hippocampal *CRH* and dopamine receptors *DRD1* and *DRD2*. Hippocampal dopamine secretion and uptake mainly originates from adrenergic neurons of the locus coeruleus (LC)^25,26^ and diffusely targets neurons in the CA1 region^19^. Hippocampus has been implicated as the main “node” in the cocaine addiction brain circuit, potentially through production of new, novel inter-neuronal circuits^27–29^. LC is also known to play an important role in driving drug addiction in response to stress based on its influence on the hippocampus and encoding of novel stimulus memories^30^. Blockade of DRD1/DRD5 receptors in hippocampus or inhibition of the LC is known to inhibit memory encoding of novel stimuli experiences^19^. Here, we suggest drug-associated memories act as behavioral reinforcement via hippocampus, producing more influence under conditions of stress in stress-sensitive individuals and leading to enhancement of drug attraction. In different phases of the addiction cycle (see below), the limbic and mesolimbic pathways have greater or lesser influence on behavior^14^. Reduction in the influence of one pathway, for example mesocortical, may allow the functionally opposing pathway (mesolimbic) more unrestricted access to influence behavioral outcome as demonstrated in studies of social defeat stress^31^. Neuroanatomical differences between stress susceptible and resilient animal models are thought to underlie differences in behavioral response to stressors^32^. We hypothesize that stress leads to increased CRH-dependent hippocampal pyramidal cell activation and reduced dopamine receptor expression producing receptor saturation and improved encoding of memories of salient stimuli in stress-prone individuals. These neurophysiological changes prime stress-prone individuals to enhanced drug use memories and increase tendency toward addictive behaviors.

The neurobiology of addiction is complex, engaging many brain structures involved to a greater or lesser extent during different phases of the addiction cycle. These stages include use (acquisition/binge), withdrawal, and preoccupation (craving). During drug exposure, the main circuits engaged are mesolimbic afferents affecting the striatum (nucleus accumbens and dorsal striatum) and thalamic nuclei^14^. Repeated acute administration of addictive drugs primes the brain reward pathways by reducing threshold for effect^33,34^, thus driving presumed neuroplasticity changes which increase responsiveness to the presence of drug. Conditioned place preference is known to be reinforced in animals with anxiety-like behavior^35^. There is mounting evidence that anti-reward circuitry is also engaged in addiction to produce aversive states as a counter balance to limbic drive^5,36–39^. In the final portion of CPP testing conducted here, mice were evaluated for preoccupation (craving) behaviors to drug, effects driven by mesolimbic circuitry possibly via the amygdala and nucleus accumbens^14,40–44^.

Hippocampal *CRH*, known to be secreted exclusively by GABAergic interneurons and thought to mainly act locally^45^, was found to be consistently upregulated in all mouse strains examined here as a result of cocaine exposure and upregulated exclusively due to stress in Sub mice. This increase in *CRH* agrees well with reports published by other authors with regards to drug exposure^46,47^ and stress, even chronic mild stress^45,48^. Drug- or stress-evoked CRH increase also parallels its memory encoding function in other brain regions, such as the shell of nucleus accumbens where the peptide has been demonstrated to act as an enhancer of cued reward responses such as return to drug use^49^, however these evoked behavioral responses are heavily dopamine release-pattern- and contextually-dependent^50^. Hippocampal CRH secretion is known to contribute to stress-induced substance abuse and increased activation of pyramidal target neurons via CRHR1, which tend to produce anxiogenic effects^51^. Upregulated *CRH* expression in socially-submissive, stress-prone mice (Sub) denotes a distinct difference in hippocampal regulation in this strain, which was also reflected in their reversal from slight aversion to cocaine to strong attraction to cocaine following CMS. This further suggests that GABA interneurons of hippocampus may impose an important stress vulnerability effect in socially submissive individuals, not only through direct action at *CRH* receptors, but also though downregulation of hippocampal dopamine receptor expression.

We report an inverse relationship between hippocampal *CRH* and *DRD1* expression, a relationship which is further exaggerated in Sub mice under stress or drug exposure, but this stress response does not appear to exist in Dom mice. Hippocampus functions in reinforcement of attraction and aversion to novel and salient stimuli, driven by DRD1/DRD5-dependent mechanisms^19,40–43,52^. Dopamine receptor downregulation as a result of stress would tend to increase salient memory encoding as a result of receptor saturation and following drug exposure would tend to reinforce drug attraction, thus aiding mesolimbic circuits driving drug use reinstatement^53–56^. Kasahara and colleagues observed this effect as well in CRH-overexpressing mice, but attributed downregulation to a system-wide compensation^23^. This may indeed be the case, since elevated CRH in Dom mice did not affect dopamine receptor expression. *CRH* strongly influences aversion and fear-conditioning (context-dependent in CA1 region of hippocampus)^57^ and produces significant regulatory effects on dopamine receptor expression exclusively in hippocampus compared against other tegmental targets such as prefrontal cortex and amygdala^23^. In socially-dominant, stress-resilient mice examined here, no change in basal hippocampal *CRH* secretion was observed, which we interpret as contributing to stress resilience.

*CRHR1* is known to be strongly linked to substance addiction^24,58^ and is a mediator of stress-induced drug attraction when activated^59,60^. It is known that CRHR1 antagonists are effective in treating addictive behaviors^61–63^. Previous studies have demonstrated activation of *CRHR1* leading to addictive behaviors occurs in mesolimbocortical targets, however hippocampal *CRHR1* is downregulated under stress^64^, as we also report here. Increased *CRH* expression under stress coupled with *CRHR1* downregulation would lead to receptor saturation, yet no increase in drug attraction was seen in Dom mice under stress conditions, which suggests the existence of compensatory mechanisms regulating stress response in this strain. Results from Sub mice suggest that observed increases in *CRH* expression and reduced *CRHR1* expression as a result of stress and accompanying reductions in dopamine receptor expression would lead to enhanced hippocampal pyramidal cell activation and enhanced encoding of stress-associated memories, respectively, and potentially sensitize stress-prone individuals under stress to addictive behaviors. This latter point is evidenced by marked increase in drug attraction for Sub mice under stress.

**Figure 5:**
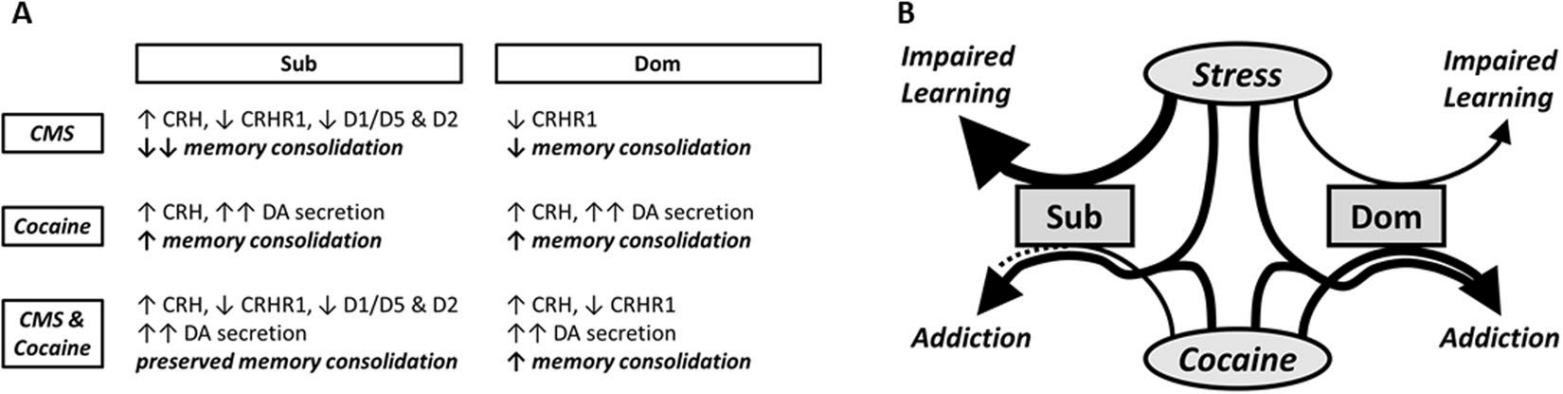
Theoretical model of neurological changes underlying differential behavioral response of Sub and Dom mouse strains. (**A)** Summary of observed and hypothesized hippocampal changes and their effects on learning. Arrows represent increase (**↑**) or decrease (**↓**). Number of arrows relates to intensity of change. (**B)** Flow diagram of cognitive and addiction-like effects of CMS and cocaine treatments. Arrow size indicates relative intensity of response. Dotted line indicates weak effect.

Our emerging model of selective and mouse strain-specific changes in hippocampus, albeit currently speculative, is presented below (Fig. 5). In Sub mice, stress increases CRH secretion which initially assists learning but through chronic application gradually results in CRHR1 downregulation^45,65,66^. This latter effect was also observed in stress-resistant Dom mice after chronic stress, however without an accompanying increase in CRH, which would reduce pyramidal neuron excitability and have some effect on memory consolidation. This result suggests downregulation of CRHR1 gene expression in Dom mice is mediated through a separate mechanism from receptor binding saturation.

Chronic stress induces a shift in LC neurons from phasic to tonic firing, also through a CRH-dependent mechanism^67–69^, which would increase norepinephrine and dopamine (DA) release in target areas such as hippocampus^30^. Increased DA release in Sub mice appears to downregulate hippocampal D1 (and presumably D5) and D2, the overall effect of which, taken in conjunction with changes in CRH stimulation, would reduce generation of long-term potentiation (LTP) in hippocampal pyramidal neurons, reduce memory consolidation, and work against drug use reinstatement. Following treatment with cocaine, Sub and Dom mice respond by increased hippocampal CRH and increased DA secretion. Cocaine is a known DA transporter antagonist^70^ and also evokes DA release through presynaptic mobilization of secondary dopamine storage pools^71^, the overall outcome of which results in increased LTP and learning relating to the stimulus, thus increasing the possibility of drug use reinstatement and drug craving.

Following application of chronic stress and treatment with cocaine, however, Sub mice show marked place preference in the CPP test, thus indicating learning and addictive-like behavior. The changes in CRH and CRHR1/DA receptor expression induced by chronic stress, which would result in reduced learning, must be compensated in some manner through increased dopamine secretion resulting from cocaine treatment, thereby preserving generation of LTP, memory consolidation, and recall of the situation in which the animal experienced the drug stimulus. In Dom mice, changes in downregulation of CRHR1 would reduce overall pyramidal cell excitability, however DA receptor expression levels are maintained, therefore stressed Dom mice given cocaine should display improved learning, yet do not increase their attraction to drug. We surmise that differences between the Dom and Sub mouse strains are mainly driven by differences in limbic function and is the subject of upcoming research. Studies to examine the cognitive effects of these neurological changes are currently underway in our laboratory.

In summary, we have demonstrated that distinct differences exist in stress susceptibility and drug attraction in mouse models exhibiting marked differences in social behavior. Previously, we reported notable differences in display of depressive-like and anxiety-like measures between Dom and Sub mice. Sub mice are socially submissive, susceptible to stress, exhibit depressive- and anxiety-like behaviors, and show a marked attraction to addictive substances when under stress. In contrast, Dom mice are socially dominant, resilient to stress, do not typically exhibit any depressive- or anxiety-like traits in behavioral tests, display a higher level of drug attraction under stress-naïve conditions and show a lack of stress-mediated increase in drug attraction. Complementing these patterns of behavior, we have observed distinct differences in hippocampal regulation of *CRH* and dopamine receptor expression paralleling response (Sub) or lack of response (Dom) to stress. Collectively, these data suggest that an underlying neurological feature of individuals that tend toward addictive behaviors may reside in personality traits and in enhanced influence of hippocampal-mediated stimulus memory neurocircuitry as a result of chronic stress.

## Methods

### Animals

Mice selectively-bred over 32 generations from a parent Sabra outbred mouse strain (Harlan Laboratories, Jerusalem, Israel) using the dominant-submissive relationship (DSR) paradigm^20,21^ has produced two animal strains distinct in several measures of social interaction and resource competition^20,21^: the dominant (Dom) and submissive (Sub) mouse strains. Wild type (WT) Sabra mice were used as a reference group. Male mice were housed in groups of five per cage except when a stress protocol dictated otherwise. Animals were given standard laboratory chow and water *ad libitum* in a colony room maintained on a 12:12 L:D cycle (lights on 07:00–19:00 hrs.). The present study conformed with NIH/USDA guidelines, and received approval from the Ariel University Institutional Animal Care and Use Committee (Permission number B7901-14-019).

### Treatment Paradigm

**Figure 6:**
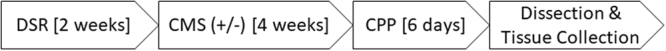
Treatment flow diagram.

For all methods, see detailed descriptions below (Fig. 6): All mice underwent dominant-submissive relationship (DSR) testing for two weeks to establish behavioral phenotype typical for each strain (Dom and Sub). In the following 4 weeks, mice were exposed either to no stressors or to a random series of stressors (chronic mild stress protocol; CMS). Next, mice underwent conditioned place preference (CPP) training and testing with either saline/saline or saline/cocaine injections within two, 2-hour windows (9:00–11:00 for saline injections and preferred-side placement, 15:00–17:00 for saline/cocaine injections and non-preferred-side placement). Following place preference testing, mice were sacrificed within 12 hours.

### Dominant-Submissive Relationship (DSR) Test

The DSR test was performed as previously^20,21^. This test was used to verify dominant (Dom strain) and submissive (Sub strain) behaviors as part of selection and colony breeding maintenance. Briefly, the DSR arena comprises two identical chambers (*l* × *w* × *h*; 12 × 8.5 × 7 cm) joined by a central, connecting tunnel (27 × 2.5 × 2.5 cm). The food target (aqueous solution of 3% milk fat, 10% sugar) is presented in the tunnel center from a subtending self-refilling well with a small access point allowing feeding by only one animal at a time. The well is bordered by removable panels to restrict access until beginning of test. DSR was conducted for 2 weeks (5 consecutive days/week). Fixed pairs of Dom and Sub mice (8-week-old, same gender, each strain) were arranged with individuals of similar weight and placed to the arena for 5 min. Milk drinking time of each animal was manually recorded.

### Chronic Mild Stress (CMS) Protocol

CMS regimen was employed based upon established protocols shown to induce anhedonia in mice^72^. Social isolation of mice is a mild stressor when the animals can still hear and smell each other from within the same room. Control mice remained in their original social housing groups (5 mice per cage), whereas stressed animals were placed in solitary housing. In a separate room, CMS mice were exposed to a randomly selected daily stressor, six days per week for four weeks: mild hunger (8 day cycle hrs., no food, water *ad libitum*), night illumination (200 lux light during the 12-hour colony dark cycle), cage tilt (8 day cycle hrs., cage angle of 30°), tail pinch (plastic clothespin was attached to the base of tail for 15 min.), forced swim (5 min., 20 cm deep, 25 °C water), cage crowding (8 day cycle hrs.: eight CMS mice were placed together in a standard cage), wet cage (250 ml water added to bedding for 8 day cycle hrs., then bedding was changed), empty cage (8 day cycle hrs, no bedding).

### Drug Injection

Cocaine (15 mg/kg i.p. in 0.9% saline) in 0.1 ml bolus.

### Conditioned Place Preference (CPP) Protocol

The CPP paradigm was used to measure addictive-like behavior^73^ by employing an apparatus, which consisted of a plastic box divided into two compartments (*l* × *w* × *h*, 17 × 15 × 37 cm; one with black and white vertical striped walls, one with black walls) with a central grey separation section (9 × 15 × 37 cm). Compartments were separated by removable dividers. Briefly, on day one mice were assessed for 20 min. without chamber dividers to determine their naïve preference to chamber. On days 2–5 (training), mice were treated twice by: a) injection with 0.9% saline and placement in the closed preferred outer compartment for 20 minutes (morning session), followed by b) injection with cocaine (15 mg/kg i.p.) and placement in the closed non-preferred outer compartment for 20 minutes (afternoon session). Time between sessions was 4 hours. On day 6 (testing), mice were placed in the closed central compartment, dividers were removed, and time spent in each chamber was recorded. All recordings were performed using EthoVision 3.1 (Noldus, Holland).

### Tissue Preparation and QT-PCR

Twelve hours after end of CPP testing, mice were sacrificed by CO_2_ asphyxiation followed by decapitation. Hippocampi were dissected, flash frozen immediately in liquid nitrogen, and stored at −80 °C until use. Quantitative PCR was performed as established previously^74,75^. Briefly, total RNA was extracted using a 5 Prime Perfect Pure RNA Tissue kit (5 Prime, USA, Cat. 2302410) and reverse transcribed to cDNAs with random primers (Promega, USA, A3500). Levels of mRNA were analyzed using a SYBR FAST Universal Readymix Kit (KAPA Biosystems, Wilmington, MA, USA) and performed in an MxPro3000 apparatus (Stratagene, Santa Clara, CA, USA). Mouse primers for target genes were designed by Integrated DNA technologies (Coralville, IA, USA) as follows: glucocorticoid receptor (GR), (F: 5′ ttctgttcatggcgtgagtacc 3′, R: 5′ cccttggcacctattccagtt 3′); corticotropin releasing hormone (*CRH*), (F: 5′ atctctctggatctcaccttcca 3′, R: 5′ atctccatcagtttcctgttgct 3′); corticotropin releasing hormone receptor type 1 (*CRHR1*), (F: 5′ ggtgtgcctttccccatcatt 3′, R: 5′ caacatgtaggtgatgcccag 3′); brain-derived neurotrophic factor (BDNF), (F: 5′ ttgttttgtgccgtttacca 3′, R: 5′ ggtaagagagccagccactg 3′); dopamine receptor 1 (*DRD1*), (F: 5′ cagaagaagaggcagcatcc 3′, R: 5′ agcaatccaagccataccag 3′); dopamine receptor 2 (*DRD2*), (F: 5′ gagaaggctttgcagaccac 3′, R: 5′ gatggcacacaggttcaaga 3′). Hypoxanthine phosphoribosyltransferase (HPRT), (F: 5′ tgttgttggatatgcccttg 3′; R: 5′ ttgcgctcatcttaggcttt 3′), gene was used as an internal control.

### Statistics

All data are expressed as mean ± SEM. Multiple comparisons were performed by one-way or two-way ANOVA, depending on experiment design, followed by a Bonferroni means separation test. Bonferroni corrections (α/m) were applied to all multiple comparisons to control for error inflation, with a threshold for significance prior to correction of α of 0.05.

## References

[1] Brady KT, McCauley JL, Back SE (2016). Prescription Opioid Misuse, Abuse, and Treatment in the United States: An Update. The American journal of psychiatry.

[2] Kolodny A (2015). The prescription opioid and heroin crisis: a public health approach to an epidemic of addiction. Annual review of public health.

[3] Russo SJ, Murrough JW, Han MH, Charney DS, Nestler EJ (2012). Neurobiology of resilience. Nature neuroscience.

[4] Sinha R (2008). Chronic stress, drug use, and vulnerability to addiction. Annals of the New York Academy of Sciences.

[5] Koob GF (2014). Addiction as a stress surfeit disorder. Neuropharmacology.

[6] Koolhaas JM (1999). Coping styles in animals: current status in behavior and stress-physiology. Neuroscience and biobehavioral reviews.

[7] Cooper MA, Clinard CT, Morrison KE (2015). Neurobiological mechanisms supporting experience-dependent resistance to social stress. Neuroscience.

[8] Koolhaas JM (2008). Coping style and immunity in animals: making sense of individual variation. Brain, behavior, and immunity.

[9] de Wit H (2009). Impulsivity as a determinant and consequence of drug use: a review of underlying processes. Addiction biology.

[10] Rappaport LM, Moskowitz DS, D’Antono B (2014). Naturalistic interpersonal behavior patterns differentiate depression and anxiety symptoms in the community. Journal of counseling psychology.

[11] Constantino MJ (2012). The relation between changes in patients’ interpersonal impact messages and outcome in treatment for chronic depression. Journal of consulting and clinical psychology.

[12] Pearson KA, Watkins ER, Mullan EG (2010). Submissive interpersonal style mediates the effect of brooding on future depressive symptoms. Behaviour research and therapy.

[13] Krebs RM, Schott BH, Duzel E (2009). Personality traits are differentially associated with patterns of reward and novelty processing in the human substantia nigra/ventral tegmental area. Biological psychiatry.

[14] Koob GF, Volkow ND (2010). Neurocircuitry of addiction. Neuropsychopharmacology: official publication of the American College of Neuropsychopharmacology.

[15] Grant S (1996). Activation of memory circuits during cue-elicited cocaine craving. Proceedings of the National Academy of Sciences of the United States of America.

[16] Prisciandaro JJ, McRae-Clark AL, Myrick H, Henderson S, Brady KT (2014). Brain activation to cocaine cues and motivation/treatment status. Addiction biology.

[17] Breiter HC (1997). Acute effects of cocaine on human brain activity and emotion. Neuron.

[18] Fotros A (2013). Cocaine cue-induced dopamine release in amygdala and hippocampus: a high-resolution PET [(1)(8)F]fallypride study in cocaine dependent participants. Neuropsychopharmacology: official publication of the American College of Neuropsychopharmacology.

[19] Takeuchi T (2016). Locus coeruleus and dopaminergic consolidation of everyday memory. Nature.

[20] Nesher E (2013). Differential responses to distinct psychotropic agents of selectively bred dominant and submissive animals. Behavioural brain research.

[21] Feder Y (2010). Selective breeding for dominant and submissive behavior in Sabra mice. J Affect Disord.

[22] Pinhasov, A., Crooke, J., Rosenthal, D., Brenneman, D. & Malatynska, E. Reduction of Submissive Behavior Model for antidepressant drug activity testing: study using a video-tracking system. *Behavioural pharmacolog*y **1**6, 657–664, doi:00008877-200512000-00009 [pii] (2005). 10.1097/00008877-200512000-00009 16286818

[23] Kasahara M, Groenink L, Olivier B, Sarnyai Z (2011). Corticotropin-releasing factor (CRF) over-expression down-regulates hippocampal dopamine receptor protein expression and CREB activation in mice. Neuro endocrinology letters.

[24] Lu L, Liu D, Ceng X (2001). Corticotropin-releasing factor receptor type 1 mediates stress-induced relapse to cocaine-conditioned place preference in rats. European journal of pharmacology.

[25] Smith CC, Greene RW (2012). CNS dopamine transmission mediated by noradrenergic innervation. The Journal of neuroscience: the official journal of the Society for Neuroscience.

[26] Borgkvist A, Malmlof T, Feltmann K, Lindskog M, Schilstrom B (2012). Dopamine in the hippocampus is cleared by the norepinephrine transporter. The international journal of neuropsychopharmacology.

[27] Ladron de Guevara-Miranda D (2017). *Long-lasting memory deficits i*n mice withdrawn from cocaine are concomitant with neuroadaptations in hippocampal basal activity, GABAergic interneurons and adult neurogenesis. Disease models & mechanisms.

[28] Castilla-Ortega E (2016). Pharmacological reduction of adult hippocampal neurogenesis modifies functional brain circuits in mice exposed to a cocaine conditioned place preference paradigm. Addiction biology.

[29] Castilla-Ortega E (2016). A place for the hippocampus in the cocaine addiction circuit: Potential roles for adult hippocampal neurogenesis. Neuroscience and biobehavioral reviews.

[30] Van Bockstaele EJ, Reyes BA, Valentino RJ (2010). The locus coeruleus: A key nucleus where stress and opioids intersect to mediate vulnerability to opiate abuse. Brain research.

[31] Chaudhury D (2013). Rapid regulation of depression-related behaviours by control of midbrain dopamine neurons. Nature.

[32] Anacker C (2016). Neuroanatomic Differences Associated With Stress Susceptibility and Resilience. Biological psychiatry.

[33] Kornetsky C, Esposito RU (1979). Euphorigenic drugs: effects on the reward pathways of the brain. Federation proceedings.

[34] Hernandez G (2006). Prolonged rewarding stimulation of the rat medial forebrain bundle: neurochemical and behavioral consequences. Behavioral neuroscience.

[35] Ladron de Guevara-Miranda D (2016). Cocaine-conditioned place preference is predicted by previous anxiety-like behavior and is related to an increased number of neurons in the basolateral amygdala. Behavioural brain research.

[36] Koob GF, Bloom FE (1988). Cellular and molecular mechanisms of drug dependence. Science.

[37] Aston-Jones G, Delfs JM, Druhan J, Zhu Y (1999). The bed nucleus of the stria terminalis. A target site for noradrenergic actions in opiate withdrawal. Annals of the New York Academy of Sciences.

[38] Nestler EJ (2001). Molecular basis of long-term plasticity underlying addiction. Nature reviews. Neuroscience.

[39] Koob GF (2003). Neuroadaptive mechanisms of addiction: studies on the extended amygdala. Eur Neuropsychopharmacol.

[40] Schott BH (2004). *Activation of midbra*in structures by associative novelty and the formation of explicit memory in humans. Learning & memory.

[41] Wittmann BC (2005). Reward-related FMRI activation of dopaminergic midbrain is associated with enhanced hippocampus-dependent long-term memory formation. Neuron.

[42] Uzakov S, Frey JU, Korz V (2005). Reinforcement of rat hippocampal LTP by holeboard training. Learning & memory.

[43] Trouche S (2016). Recoding a cocaine-place memory engram to a neutral engram in the hippocampus. Nature neuroscience.

[44] Rosen ZB, Cheung S, Siegelbaum SA (2015). Midbrain dopamine neurons bidirectionally regulate CA3-CA1 synaptic drive. Nature neuroscience.

[45] Chen Y (2004). Hippocampal corticotropin releasing hormone: pre- and postsynaptic location and release by stress. Neuroscience.

[46] Guan X, Wan R, Zhu C, Li S (2014). Corticotropin-releasing factor receptor type-2 is involved in the cocaine-primed reinstatement of cocaine conditioned place preference in rats. Behavioural brain research.

[47] Jakovcevski M, Schachner M, Morellini F (2011). Susceptibility to the long-term anxiogenic effects of an acute stressor is mediated by the activation of the glucocorticoid receptors. Neuropharmacology.

[48] Regev L, Baram TZ (2014). Corticotropin releasing factor in neuroplasticity. Frontiers in neuroendocrinology.

[49] Pecina S, Schulkin J, Berridge KC (2006). Nucleus accumbens corticotropin-releasing factor increases cue-triggered motivation for sucrose reward: paradoxical positive incentive effects in stress?. BMC biology.

[50] Chen YW, Rada PV, Butzler BP, Leibowitz SF, Hoebel BG (2012). Corticotropin-releasing factor in the nucleus accumbens shell induces swim depression, anxiety, and anhedonia along with changes in local dopamine/acetylcholine balance. Neuroscience.

[51] Haass-Koffler CL, Bartlett SE (2012). Stress and addiction: contribution of the corticotropin releasing factor (CRF) system in neuroplasticity. Frontiers in molecular neuroscience.

[52] Kramar CP, Barbano MF, Medina JH (2014). Dopamine D1/D5 receptors in the dorsal hippocampus are required for the acquisition and expression of a single trial cocaine-associated memory. Neurobiology of learning and memory.

[53] Shaham Y, Shalev U, Lu L, De Wit H, Stewart J (2003). The reinstatement model of drug relapse: history, methodology and major findings. Psychopharmacology (Berl).

[54] Shalev U, Grimm JW, Shaham Y (2002). Neurobiology of relapse to heroin and cocaine seeking: a review. Pharmacological reviews.

[55] Lodge DJ, Grace AA (2005). Acute and chronic corticotropin-releasing factor 1 receptor blockade inhibits cocaine-induced dopamine release: correlation with dopamine neuron activity. The Journal of pharmacology and experimental therapeutics.

[56] Castro DC, Cole SL, Berridge KC (2015). Lateral hypothalamus, nucleus accumbens, and ventral pallidum roles in eating and hunger: interactions between homeostatic and reward circuitry. Frontiers in systems neuroscience.

[57] Radulovic J, Ruhmann A, Liepold T, Spiess J (1999). Modulation of learning and anxiety by corticotropin-releasing factor (CRF) and stress: differential roles of CRF receptors 1 and 2. The Journal of neuroscience: the official journal of the Society for Neuroscience.

[58] Silberman Y (2013). & Winder, D. G. Emerging role for corticotropin releasing factor signaling in the bed nucleus of the stria terminalis at the intersection of stress and reward. Frontiers in psychiatry.

[59] Cleck JN, Blendy JA (2008). Making a bad thing worse: adverse effects of stress on drug addiction. The Journal of clinical investigation.

[60] Kreibich AS (2009). Stress-induced potentiation of cocaine reward: a role for CRF R1 and CREB. Neuropsychopharmacology: official publication of the American College of Neuropsychopharmacology.

[61] Contoreggi C, Rice KC, Chrousos G (2004). Nonpeptide corticotropin-releasing hormone receptor type 1 antagonists and their applications in psychosomatic disorders. Neuroendocrinology.

[62] Logrip ML, Koob GF, Zorrilla EP (2011). Role of corticotropin-releasing factor in drug addiction: potential for pharmacological intervention. CNS drugs.

[63] Molander A (2012). Brain-specific inactivation of the Crhr1 gene inhibits post-dependent and stress-induced alcohol intake, but does not affect relapse-like drinking. Neuropsychopharmacology: official publication of the American College of Neuropsychopharmacology.

[64] Greetfeld M (2009). A single episode of restraint stress regulates central corticotrophin- releasing hormone receptor expression and binding in specific areas of the mouse brain. J Neuroendocrinol.

[65] Chen Y, Andres AL, Frotscher M, Baram TZ (2012). Tuning synaptic transmission in the hippocampus by stress: the CRH system. Frontiers in cellular neuroscience.

[66] Brunson KL, Grigoriadis DE, Lorang MT, Baram TZ (2002). Corticotropin-releasing hormone (CRH) downregulates the function of its receptor (CRF1) and induces CRF1 expression in hippocampal and cortical regions of the immature rat brain. Experimental neurology.

[67] Kosoyan HP, Grigoriadis DE, Tache Y (2005). The CRF(1) receptor antagonist, NBI-35965, abolished the activation of locus coeruleus neurons induced by colorectal distension and intracisternal CRF in rats. Brain research.

[68] Lechner SM, Curtis AL, Brons R, Valentino RJ (1997). Locus coeruleus activation by colon distention: role of corticotropin-releasing factor and excitatory amino acids. Brain research.

[69] Valentino RJ, Page ME, Curtis AL (1991). Activation of noradrenergic locus coeruleus neurons by hemodynamic stress is due to local release of corticotropin-releasing factor. Brain research.

[70] Nestler EJ (2005). The neurobiology of cocaine addiction. Science & practice perspectives.

[71] Venton BJ (2006). Cocaine increases dopamine release by mobilization of a synapsin-dependent reserve pool. The Journal of neuroscience: the official journal of the Society for Neuroscience.

[72] Gross M, Pinhasov A (2016). Chronic mild stress in submissive mice: Marked polydipsia and social avoidance without hedonic deficit in the sucrose preference test. Behavioural brain research.

[73] Prus, A. J., James, J. R. & Rosecrans, J. A. in *Methods of Behavior Analysis in Neuroscience Frontiers in Neuroscience* (ed J. J. Buccafusco) (2009).

[74] Schechter M (2013). Endocannabinoid receptor deficiency affects maternal care and alters the dam’s hippocampal oxytocin receptor and brain-derived neurotrophic factor expression. J Neuroendocrinol.

[75] Pinhasov A (2005). Different levels of gamma-synuclein mRNA in the cerebral cortex of dominant, neutral and submissive rats selected in the competition test. Genes, brain, and behavior.

